# Functional and Evolutionary Insights of NR1J1 Nuclear Receptors: A Diversified Sensing Weapon in Bivalves

**DOI:** 10.3390/ijms27156810

**Published:** 2026-07-29

**Authors:** Maria Paula Gómez-Román, Elza Fonseca, Mário Jorge Araújo, Vitor Vasconcelos, Alexandre Campos

**Affiliations:** 1 CIIMAR/CIMAR LA, Centro Interdisciplinar de Investigação Marinha e Ambiental, Universidade do Porto, Terminal de Cruzeiros do Porto de Leixões, 4450-208 Matosinhos, Portugal; mgomez@ciimar.up.pt (M.P.G.-R.); efonseca@ciimar.up.pt (E.F.); mario.araujo@ciimar.up.pt (M.J.A.); vmvascon@fc.up.pt (V.V.); 2 ICBAS, Instituto de Ciências Biomédicas de Abel Salazar, Universidade do Porto, Rua de Jorge Viterbo Ferreira 228, 4050-313 Porto, Portugal; 3 Departamento de Biologia, Faculdade de Ciências, Universidade do Porto, Rua Campo Alegre s/n, 4169-007 Porto, Portugal

**Keywords:** transcription factors, xenobiotic sensing, functional responses, evolutionary diversification, ligand responsiveness, *Mytilus galloprovincialis*, *Ruditapes decussatus*

## Abstract

Nuclear receptors (NRs) of the NR1J1 group have been described in aquatic invertebrates and proposed as the evolutionary counterparts of vertebrate NR1I receptors, known for their role in xenobiotic-sensing and detoxification. In bivalves, previous studies have shown that NR1J1 activity and gene expression are modulated by pharmaceuticals, toxins, natural compounds, and algal extracts. However, their functional properties and diversification remain poorly understood. Here, we investigated the function and evolution of NR1J1 receptors in the bivalves *Mytilus galloprovincialis* and *Ruditapes decussatus*. We first identified four *nr1j1* paralogs in *R. decussatus* and then integrated phylogenetic analysis, domain identity comparisons, tissue distribution profiling, and luciferase-based transactivation assays to characterize NR1J1 paralogs from both species. The receptors displayed distinct but partially overlapping transactivation profiles in response to confirmed ligands of NR1I and NR1H receptors, natural compounds, plant extracts, and fish bile, indicating paralog- and species-specific differences in ligand responsiveness. Allocholic acid emerged as the most consistent agonist across paralogs, whereas curcumin, carnosic acid, and *Ptychopetalum olacoides* extract showed more selective response patterns. Tissue profiling revealed broad but non-uniform expression of *nr1j1* paralogs across bivalve tissues. Together, these findings underscore that bivalve NR1J1 receptors constitute a functionally diversified chemical-sensing system and provide a framework for future studies addressing ligand-dependent regulation and detoxification pathways.

## 1. Introduction

Bivalve mollusks are aquatic predominantly filter-feeding invertebrates that continuously process large volumes of water and, consequently, accumulate a broad range of exogenous compounds, including marine toxins, pathogenic microorganisms, heavy metals, nanoplastics, and other environmental pollutants [1,2,3,4]. In addition to their ecological relevance in water quality improvement, nutrient cycling, and bioremediation, their filter-feeding capacity also makes bivalves important sentinels of environmental contamination and potential vectors of toxic compounds to human consumers [5,6,7]. Currently, depuration is the only approved strategy to reduce microbial contamination in bivalves, and its limited effectiveness in removing biotoxins and other chemical contaminants restricts its broader application [8,9]. Therefore, identifying the molecular mechanisms that govern chemical sensing, metabolism, and detoxification in bivalves is of major interest for both environmental and seafood safety perspectives.

In animals, the detection, metabolism, and elimination of endogenous and exogenous chemicals depend on the chemical defensome, a coordinated network comprising ligand-activated transcription factors, drug-metabolizing enzymes, and membrane transporters [10,11,12]. Among these components, nuclear receptors (NRs) are especially relevant because they regulate the expression of genes involved in a variety of biological functions, including metabolism, development, reproduction, immunity, and stress responses [13,14]. In vertebrates, the NR1I group, particularly the pregnane X receptor (PXR; NR1I2) and the constitutive androstane receptor (CAR; NR1I3), has been extensively studied as a central regulatory core in xenobiotic sensing and detoxification [10,11]. These NRs are activated by a chemically diverse range of compounds, including pharmaceuticals, steroid hormones, dietary elements, and environmental pollutants [15,16,17,18,19], and subsequently regulate genes involved in the biotransformation and detoxification of exogenous and endogenous compounds [20,21,22,23,24]. Previous phylogenetic analyses have placed protostome NR1J receptors within the broader NR1I-like NR radiation, although direct one-to-one orthology with individual vertebrate NR1I receptors cannot be inferred [25,26,27,28]. Members of the NR1J1 group have been identified in several invertebrates and proposed to participate in metabolism- and detoxification-related processes [25,26,27,28]. However, in contrast to vertebrate NR1I receptors, the ligand responsiveness, functional properties, and evolutionary diversification of NR1J1 receptors remain poorly understood. This gap is particularly relevant in bivalves, where multiple NR1J1 paralogs have been reported, raising the possibility that receptor diversification may have been accompanied not only by functional specialization in ligand recognition and transcriptional responses, but also by differences in domain conservation and tissue distribution. In this context, comparing the conservation of functional domains and the tissue distribution of NR1J1 paralogs may help to clarify whether receptor diversification in bivalves is associated with conserved DNA recognition features, divergence in ligand-related properties, and potential subfunctionalization among paralogs.

To date, functional information on bivalve NR1J1 receptors remains limited. In the Pacific oyster *Crassostrea gigas* (Thunberg, 1793), *nr1j1* paralogs were shown to display differential expression across developmental stages and adult life stages [29]. In the peppery furrow shell *Scrobicularia plana* (da Costa, 1778), the modulation of an NR1J1 paralog was altered following exposure to the marine toxin okadaic acid and pesticides esfenvalerate and triclosan, suggesting its potential involvement in xenobiotic-sensing and detoxification response pathways [26]. In the Mediterranean mussel *Mytilus galloprovincialis* (Lamarck, 1819), the expression of three *nr1j1* paralogs and candidate downstream genes were altered in response to pharmaceuticals warfarin, dexamethasone, and imidazole [27]. The activity of the four mussel NR1J1 receptors was modulated by freshwater cyanotoxins, marine toxins, non-toxic natural compounds, and algal extracts [30], while recent in vivo evidence further suggested that the expression of *nr1j1* paralogs and candidate downstream target genes are altered following exposure to the microalga *Tetraselmis* sp. [28]. Collectively, these studies support the view that NR1J1 receptors participate in bivalve chemical response pathways. However, they do not clarify how distinct NR1J1 paralogs differ in their functional and evolutionary relationship, domain conservation, tissue distribution, and functional responses to structurally diverse ligands. Natural compounds are of particular interest as candidate modulators of detoxification-related pathways. In humans, several natural molecules, including phytochemicals, have been reported to activate PXR and modulate detoxification-related pathways, with implications for metabolism and inflammation, establishing this receptor as a therapeutic target and a key factor to understand herb-drug interactions [31,32,33,34,35]. In bivalves, compounds such as curcumin and cinnamaldehyde have been associated with altered expression of detoxification-related genes and reduced accumulation of diarrhetic shellfish toxins [36,37]. Nevertheless, whether natural compounds and other non-toxic molecules can directly modulate the activity of bivalve NR1J1 receptors, and whether such responses are associated with paralog-specific patterns of conservation and expression, remains largely unexplored. Addressing this question is important not only to improve understanding of NR1J1 receptor function but also to identify candidate compounds with potential to modulate detoxification-associated responses in shellfish.

Accordingly, we hypothesized that NR1J1 paralogs from two bivalve species exhibit comparable transcriptional responses to chemically diverse compounds, sharing similar roles in xenobiotic metabolism. To test this hypothesis, the present study aimed to characterize the functional and evolutionary diversification of NR1J1 receptors in the epifaunal Mediterranean mussel *M. galloprovincialis* and the infaunal European clam *Ruditapes decussatus* (Linnaeus, 1758). These two species occupy different habitats and are therefore exposed to different environmental conditions, making them relevant for comparative analyses of NR1J1 receptors [38,39]. For this purpose, we first isolated four *nr1j1* paralogs from *R. decussatus* and examined their evolutionary relationships with homologous receptors from other metazoans. We additionally compared sequence conservation across functional domains and assessed the tissue distribution of *nr1j1* paralogs in both bivalve species. Then, we assessed the transactivation of eight NR1J1 receptor paralogs from both bivalve species in response to a chemically diverse panel of compounds, including confirmed ligands of vertebrate NR1I and NR1H receptors, natural compounds, plant extracts and fish bile. By integrating phylogenetic, domain identity, expression, and transactivation data, this study presents the first comparative characterization of NR1J1 receptors from *M. galloprovincialis* and *R. decussatus*, including their tissue expression patters and functional responses to a chemically diverse panel of compounds. These findings provide new insights into the functional diversity of bivalve NR1J1 receptors and their potential involvement in chemical-sensing and detoxification-related pathways.

## 2. Results

### 2.1. Identification and Tissue Distribution of NR1J1 Paralogs in Bivalves

Our searches in *R. decussatus* transcriptomes from the Sequence Read Archive (SRA) data identified partial sequences corresponding to four different genes. The full open-reading frames were isolated and identified as the four *nr1j1* paralogs of *R. decussatus*, namely Rde*nr1j1α*, Rde*nr1j1β*, Rde*nr1j1γ*, and Rde*nr1j1δ*. Together with the previously reported *M. galloprovincialis* NR1J1 counterparts and other mollusks, these data support a molluscan NR1J repertoire, composed of up to four retained paralogs depending on the lineages [27,29,40]. To establish the evolutionary relationships of *nr1j1* paralogs in *M. galloprovincialis* and *R. decussatus*, a phylogenetic analysis of amino acid sequences was first performed.

**Figure 1:**
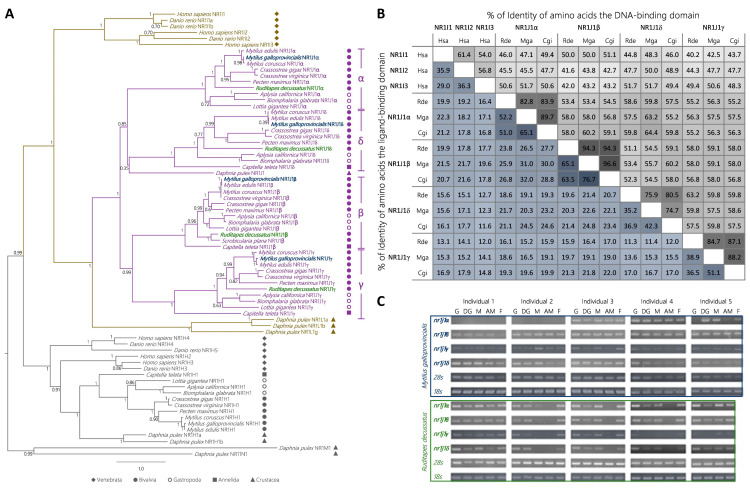
Evolutionary and expression overview of *M. galloprovincialis* and *R. decussatus* NR1J1 receptors. (**A**) Maximum likelihood phylogenetic tree describing relationships among NR1J1 (purple), vertebrate NR1I (yellow), and crustacean NR1L (yellow). NR1H, NR1M, and NR1N receptors used as outgroup (gray). Blue and green colors indicate NR1J1 paralogs from *M. galloprovincialis* and *R. decussatus*, respectively. The analysis was conducted using aBayes interference. The numbers on the nodes represent aBayes branch-support values. (**B**) Identity matrix with the percentage of identical amino acids in the DNA-binding (gray gradient) and ligand-binding (blue gradient) domains between *Homo sapiens* (Hsa) NR1I members and bivalves *R. decussatus* (Rde), *M. galloprovincialis* (Mga), and *C. gigas* (Cgi) NR1J members. (**C**) Expression pattern of *nr1j1* paralogs in tissue panels of five individuals of *M. galloprovincialis* and *R. decussatus* using endpoint PCR. Times of 28s and 18s were used as housekeeping genes. G: gill, DG: digestive gland, M: mantle, AM: adductor muscle, F: foot. The adductor muscle sample from individual 5 of *R. decussatus* was excluded because RNA yield was insufficient for analysis.

The resulting tree resolved four main NR1J1 clades (α, β, γ, and δ), in which the sequences from both bivalve species of interest clustered with homologous sequences from the corresponding NR1J1 paralog clades in other mollusks (Figure 1A). NR1J1 sequences were clearly separated from the NR1H clade and from crustacean NR1N and NR1M receptors, while remaining within the cluster of vertebrate NR1I and crustacean NR1L, consistent with an ancient evolutionary relationship among these receptors. The identity matrix further showed that the DNA-binding domain (DBD) is more conserved than the ligand-binding domain (LBD) when comparing human NR1I and bivalve NR1J1 receptors. Orthologous bivalve NR1J1 paralogs showed higher within-clade similarity. Interestingly, LBDs of bivalve NR1J1 paralogs showed more similarity to human NR1I1 (vitamin D receptor; VDR) and NR1I2 (PXR) receptors, while the DBDs showed differential sequence similarity to human NR1I receptors, with NR1J1α-DBD more similar to human NR1I3 (CAR), NR1J1β-DBD to NR1I1, and NR1J1γ/δ-DBDs to both NR1I2 and NR1I3 (Figure 1B). Despite the overall conservation of the DBD, paralog-specific divergence is also detectable in the DNA-recognition module, whereas the LBD retains a conserved NR1I-like similarity pattern. Tissue profiling detected *nr1j1* transcripts in all examined tissues of both bivalve species, although with paralog- and individual-dependent variation in signal intensity (Figure 1C), supporting a broad but non-uniform distribution of these receptors among tissues.

### 2.2. Functional Screening of Confirmed Ligands of Vertebrate NR1I and NR1H Receptors on NR1J1 Receptors from M. galloprovincialis and R. decussatus

**Figure 2:**
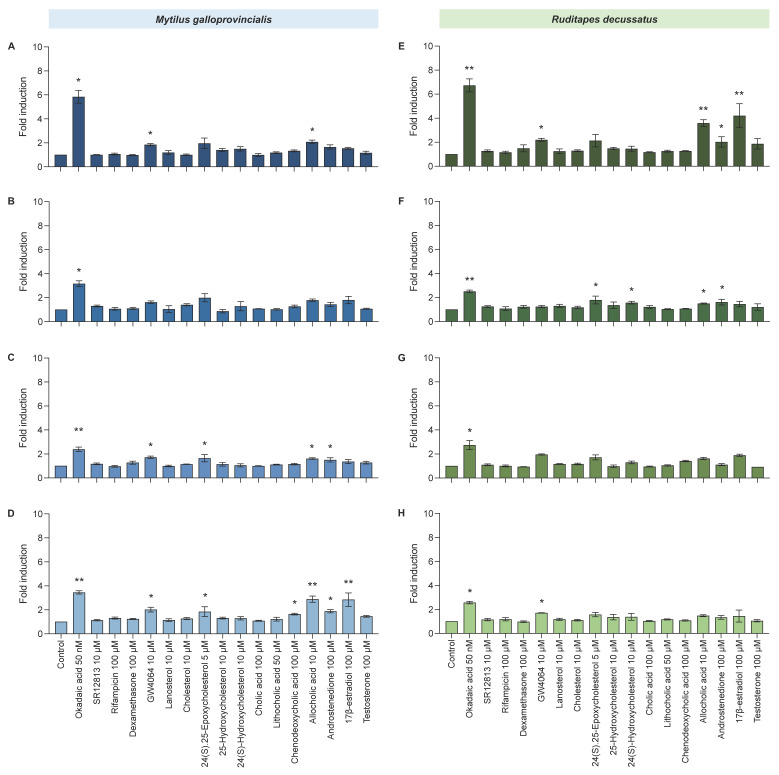
Transactivation responses of (**A**) MgaNR1J1α, (**B**) MgaNR1J1β, (**C**) MgaNR1J1γ, (**D**) MgaNR1J1δ, (**E**) RdeNR1J1α, (**F**) RdeNR1J1β, (**G**) RdeNR1J1γ, and (**H**) RdeNR1J1δ following exposure to confirmed ligands of vertebrate NR1I and NR1H receptors, including synthetic ligands, an antibiotic, a corticosteroid, lanosterol, cholesterol and its derivatives such as oxysterols, bile acids and steroid hormones. Significant differences relative to the DMSO control are represented by * q-value < 0.05, ** q-value < 0.01. Data are presented as mean ± standard error of the mean (n = 3).

Based on the evolutionary relationship between NR1J, NR1I, and NR1H groups, we evaluated whether compounds known to activate NR1I and NR1H receptors were also able to modulate the activity of bivalve NR1J1 paralogs. The selected panel included synthetic ligands, antibiotic, corticosteroid, lanosterol, cholesterol and its derivatives, such as oxysterols, bile acids, and steroid hormones (Supplementary Table S1). COS-1 cells, transfected with plasmids encoding the NR1J1 paralogs from both bivalve species and the human PXR, were subjected to a dual-luciferase transactivation assay following exposure to the selected compounds. As NR1J1 activation profiles remain poorly characterized, human PXR responses were included as a vertebrate reference, and results are shown in Supplementary Figure S1. Okadaic acid, used as a positive control, significantly activated all MgaNR1J1 and RdeNR1J1 paralogs, with fold inductions of 5.8, 3.2, 2.4, and 3.5 in MgaNR1J1α, β, γ, and δ paralogs (Figure 2A–D) and 6.7, 2.5, 2.7, and 2.6 in RdeNR1J1 α, β, γ, and δ paralog receptors (Figure 2E–H). Based on the fold induction, the effects of the tested compounds were classified as potent activators (≥4-fold induction), strong activators (≥3 to <4-fold induction), moderate activators (≥2 to <3-fold induction), weak or non-activators (<2-fold induction), and potential inverse agonists (<0.6-fold induction).

Overall, the tested compounds led to distinct transactivation responses across MgaNR1J1 and RdeNR1J1 paralogs. Among mussel receptors, MgaNR1J1δ (Figure 2D) showed the broadest responsiveness, reaching 2.9-fold induction with allocholic acid and 17β-estradiol, 2-fold induction with GW4046, and 1–8-fold induction with 24(S),25-epoxycholesterol and androstenedione, whereas MgaNR1J1β (Figure 2B) and MgaNR1J1γ (Figure 2C) were overall least responsive, with several compounds showing weak or non-activation effect. Among the clam receptors, RdeNR1J1α (Figure 2E) showed the broadest overall responsiveness, a 4.2-fold activation with 17β-estradiol, 3.6-fold activation with allocholic acid, 2.2-, 2.1-, and 2-fold activation with GW4064, 24(S),25-epoxycholesterol, and androstenedione, respectively, whereas RdeNR1J1β (Figure 2F), RdeNR1J1γ (Figure 2G), and RdeNR1J1δ (Figure 2H) were less responsive across most treatments.

Several compounds previously associated with vertebrate NR1I and NR1H signaling also activated multiple NR1J1 paralogs in both species. Allocholic acid was the compound with the broadest activation profile, inducing weak to strong activation ranging from 1.6- to 2.9-fold and 1.5- to 3.6-fold in MgaNR1J1 and RdeNR1J1 paralogs, respectively. Likewise, 24(S),25-epoxycholesterol and GW4064 induced weak or non-activation to moderate activation, with fold inductions between 1.2 to 2.1 in both mussel and clam paralogs. Dexamethasone, chenodeoxycholic acid, 24(S)-hydroxycholesterol, rifampicin, SR12813, lanosterol, cholesterol, cholic acid, and lithocholic acid were weak or non-activators of all receptors, generally remaining close to control values. Steroid hormones also elicited differential responses across NR1J1 paralogs. In both bivalve species, 17β-estradiol produced the strongest hormone-related response, particularly in RdeNR1J1α (4.2-fold) and MgaNR1J1δ (2.9-fold), followed by androstenedione, which induced a moderate activation (2.0-fold) of RdeNR1J1α paralog. Conversely, testosterone showed weak or non-activation in all paralogs.

### 2.3. Effect of Selected Natural Compounds and Plant Extracts on Activation Responses of NR1J1 Receptors from M. galloprovincialis and R. decussatus

Given the reported involvement of NR1J1 receptors in bivalve responses to marine toxins and other stressors [27,28,30], we next assessed the ability of natural compounds and plant extracts to modulate NR1J1 transactivation. In contrast to the compounds evaluated above, these compounds were selected as non-toxic or less-toxic candidates with potential biotechnological relevance for the development of strategies to improve detoxification responses in bivalves. Transactivation responses of MgaNR1J1 receptors were first assessed by a broad primary screening followed by a dose–response assay, after which the most active compounds were evaluated in RdeNR1J1 receptors.

#### 2.3.1. Transactivation Responses of NR1J1 Receptors from *M. galloprovincialis*

**Figure 3:**
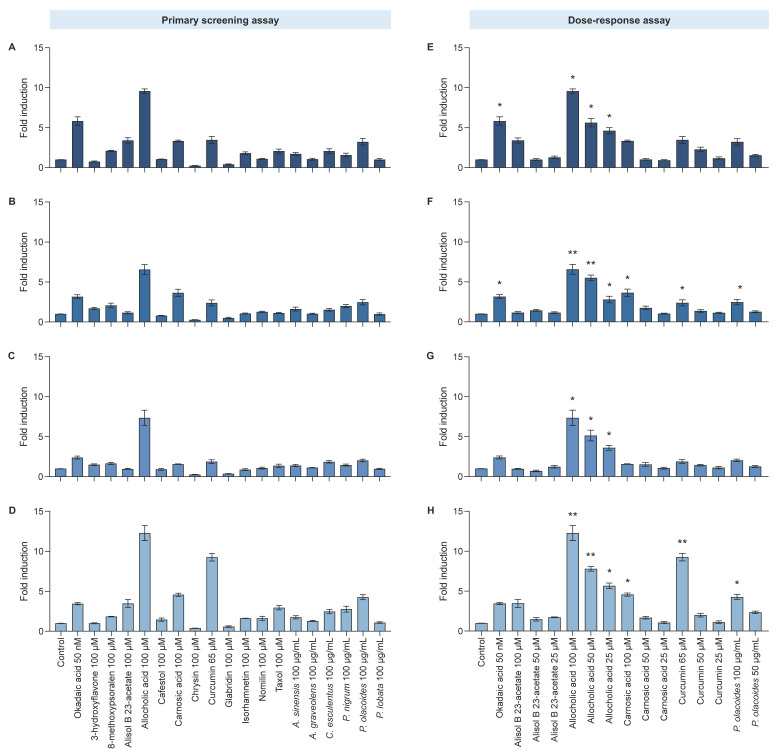
Screening and dose–response evaluation of 12 natural compounds and 6 plant extracts on *M. galloprovincialis* NR1J1 paralogs. Primary screening responses are shown for (**A**) MgaNR1J1α, (**B**) MgaNR1J1β, (**C**) MgaNR1J1γ, and (**D**) MgaNR1J1δ after exposure to the selected compounds at the highest tested concentration. Dose–response effects are shown for (**E**) MgaNR1J1α, (**F**) MgaNR1J1β, (**G**) MgaNR1J1γ, and (**H**) MgaNR1J1δ after exposure to selected active compounds and plant extracts at the indicated concentrations. Asterisks indicate significant differences in fold induction compared with the DMSO control (* q-value < 0.05, ** q-value < 0.01). Data are presented as mean ± standard error of the mean (n = 3).

We first evaluated the responsiveness of MgaNR1J1 receptors to 12 natural compounds (100 μM) and 6 plant extracts (100 μg/mL) using a primary screening assay. As curcumin reduced COS-1 cells viability at 100 and 75 μM, but not at 65 μM, the former was used as its highest concentration. Results are shown in Figure 3A–D and Supplementary Table S1.

Among the 18 tested conditions, allocholic acid displayed the highest and broadest response across the four MgaNR1J1 paralogs, inducing 9.6-, 6.6-, 7.3-, and 12.3-fold activation in MgaNR1J1α, β, γ, and δ, respectively. Carnosic acid and curcumin displayed a similar activation pattern, with moderate to potent paralog-dependent activity in MgaNR1J1α (3.3- to 3.5-fold), MgaNR1J1β (2.4- to 6.6-fold), and MgaNR1J1δ (4.6- to 9.3-fold). In contrast, weak or no-activation was observed in MgaNR1J1γ (1.6- to 1.9-fold). *Ptychopetallum olacoides* extract also activated all four paralogs, with 3.2-, 2.5-, 2.0-, and 4.3-fold induction, indicating moderate to potent agonist activity, with the strongest response in MgaNR1J1δ. Alisol B 23-acetate showed strong activity on MgaNR1J1α and MgaNRI1J1δ, with 3.4- and 3.5-fold induction, respectively, whereas MgaNR1J1β and MgaNR1J1γ remained near control levels. Moderate activation of MgaNR1J1α, MgaNR1J1β, or MgaNR1J1δ was induced by 8-methoxypsoralen, taxol, *Cyperus esculentus* extract or *Piper nigrum* extract, with activation levels between 2.0- to 2.9-fold induction, whereas weaker responses were observed for MgaNR1J1γ. In contrast, chrysin and glabridin consistently reduced reporter activity relative to the solvent control across all paralogs. Chrysin induced 0.2-, 0.3-, 0.3-, and 0.4-fold activity in MgaNR1J1α, β, γ, and δ, respectively, whereas glabridin induced 0.4-, 0.5-, 0.4-, and 0.6-fold activity. These profiles identify both compounds as candidate negative modulators of MgaNR1J1 activity under the present assay conditions. The remaining compounds showed weak or no agonist activity in all mussel NR1J1 paralogs.

Compounds that induced strong to potent activation (≥3-fold) in at least one MgaNR1J1 paralog during the primary screening were subsequently selected for dose–response assays. Thus, transfected COS-1 cells were exposed to alisol B 23-acetate, allocholic acid, carnosic acid, curcumin, and *P. olacoides* extract at lower concentrations (Figure 3E–H and Supplementary Table S1). Allocholic acid remained the most active compound across the four MgaNR1J1 paralogs and consistently showed the most stable activation profile among the evaluated compounds. This compound retained strong to potent activation at 50 μM and 25 μM in the MgaNR1J1α (5.6- and 4.6-fold), MgaNR1J1γ (5.1- and 3.6-fold), and MgaNR1J1δ (7.8- and 5.7-fold) paralogs, while potent activation in MgaNR1J1β (5.5-fold) only at 50 μM. In contrast, curcumin displayed a more selective and concentration-dependent pattern. Although MgaNR1J1α, MgaNR1J1β, and MgaNR1J1δ reached moderate to strong activation at 65 μM, moderate activation was observed at 50 μM in only MgaNR1J1α and MgaNR1J1δ paralogs. Finally, in MgaNR1J1γ, curcumin induced approximately 1.9-fold, 1.4-fold, and 1.1-fold at 65, 50, and 25 μM, respectively, indicating weak or non-activation across all tested concentrations. The remaining compounds showed narrower dose–response profiles, exhibiting weak or no changes in luciferase activity on all paralogs.

#### 2.3.2. Transactivation Responses of NR1J1 Receptors from *R. decussatus*

**Figure 4:**
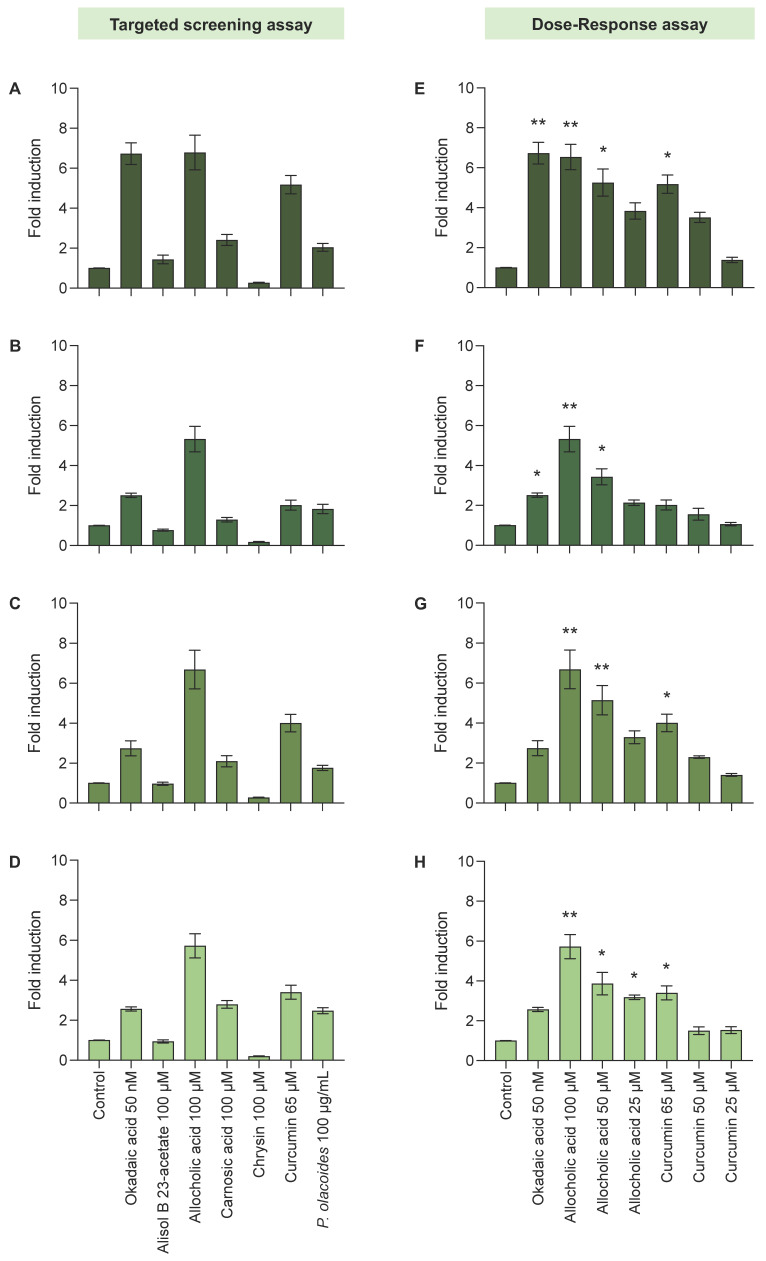
Targeted screening and dose–response evaluation of selected natural compounds and plant extracts on *R. decussatus* NR1J1 paralogs. Targeted screening responses are shown for (**A**) RdeNR1J1α, (**B**) RdeNR1J1β, (**C**) RdeNR1J1γ, and (**D**) RdeNR1J1δ after exposure to selected natural compounds and plant extracts at the highest tested concentration. Dose–response effects are shown for (**E**) RdeNR1J1α, (**F**) RdeNR1J1β, (**G**) RdeNR1J1γ, and (**H**) RdeNR1J1δ after exposure to selected active compounds at the indicated concentrations. Asterisks indicate significant differences in fold induction compared with the DMSO control (* q-value < 0.05, ** q-value < 0.01). Data are presented as mean ± standard error of the mean (n = 3).

Based on the primary screening results obtained for MgaNR1J1 paralogs, we performed a targeted screening in RdeNR1J1 paralogs using the four compounds and the plant extract that showed strong to potent agonist activity (≥3-fold activation) in at least one MgaNR1J1 paralog. Chrysin was also included to assess whether the reduction in reporter activity observed in MgaNR1J1 receptors was conserved in *R. decussatus* (Figure 4A–D and Supplementary Table S1).

Allocholic acid was again the most active compound, inducing potent activation with 6.5-fold, 5.3-fold, 6.7-fold, and 5.7-fold in RdeNR1J1α, β, γ, and δ, respectively. Curcumin showed moderate to potent agonist activity, with inductions of 5.2-fold in RdeNR1J1α, 4.0-fold in RdeNR1J1γ, and 3.4-fold in RdeNR1J1δ, whereas the response in RdeNR1J1β remained lower, at 2.0-fold. The *P. olacoides* extract displayed weaker activity, reaching 2.0-fold in RdeNR1J1α and 2.5-fold in RdeNR1J1δ, while alisol B 23-acetate remained close to control levels in all four paralogs. Similar to the pattern observed in MgaNR1J1 receptors, chrysin reduced reporter activity across all RdeNR1J1 paralogs to 0.2–0.3-fold, indicating a conserved negative modulatory effect between the two bivalve species.

As allocholic acid and curcumin were the only compounds showing strong to potent agonist activity in at least one RdeNR1J1 paralog, they were selected for dose–response assays (Figure 4E–H and Supplementary Table S1). Allocholic acid remained the strongest and most consistent activator, maintaining moderate to strong activation in all RdeNR1J1 paralogs at both 50 and 25 μM, with induction levels reaching approximately 5.3- and 3.8-fold in RdeNR1J1α, 3.4- and 2.1-fold in RdeNR1J1β, 5.1- and 3.3-fold in RdeNR1J1γ, and 3.9- and 3.2-fold in RdeNR1J1δ, respectively. Conversely, curcumin displayed a more selective response profile. RdeNR1J1α and RdeNR1J1γ showed 3.5- and 2.3-fold induction at 50 μM, respectively, whereas approximately 1.4-fold was observed at 25 μM in both paralogs. In contrast, RdeNR1J1β and RdeNR1J1δ exhibited weak or non-activation, with inductions ranging from 1.1- to 1.6-fold at 25 and 50 μM.

### 2.4. Effect of Bile Acids on Activation Responses of NR1J1 Receptors from M. galloprovincialis and R. decussatus

**Figure 5:**
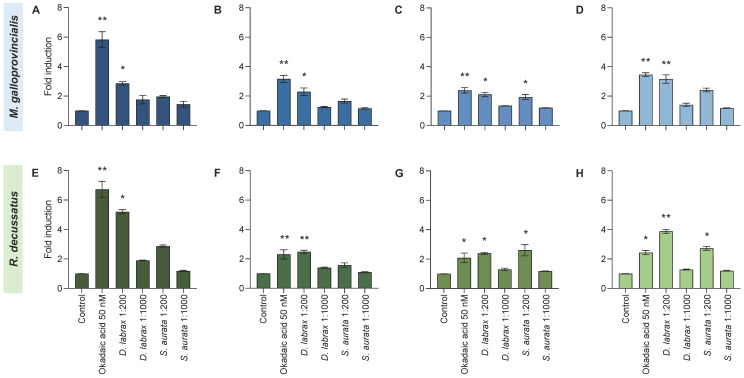
Dose–response effects of fish bile samples on NR1J1 paralogs from *M. galloprovincialis* and *R. decussatus*. Responses of *M. galloprovincialis* paralogs are shown for (**A**) MgaNR1J1α, (**B**) MgaNR1J1β, (**C**) MgaNR1J1γ, and (**D**) MgaNR1J1δ, whereas responses of *R. decussatus* paralogs are shown for (**E**) RdeNR1J1α, (**F**) RdeNR1J1β, (**G**) RdeNR1J1γ, and (**H**) RdeNR1J1δ after exposure to bile samples from *Dicentrarchus labrax* and *Sparus aurata* at 1:200 and 1:1000 dilutions. Asterisks indicate significant differences in fold induction compared with the DMSO control (* q-value < 0.05, ** q-value < 0.01). Data are presented as mean ± standard error of the mean (n = 3).

After identifying allocholic acid as the most consistent activator of NR1J1 receptors in both bivalve species, we investigated whether related activity could also be detected in crude fish bile samples given their sterol-rich content [41,42,43]. Bile samples collected from the gall bladder of European seabass *Dicentrarchus labrax* and Gilthead seabream *Sparus aurata* showed relative protein concentrations of 0.15 and 0.32 µg/mL, respectively, at 1:1000 dilution, using a BSA standard curve. These samples were then tested in a dose–response assay, using 1:200 and 1:1000 dilutions, to assess their ability to activate MgaNR1J1 and RdeNR1J1 receptors (Figure 5 and Supplementary Table S1).

In *M. galloprovincialis* (Figure 5A–D), *D. labrax* bile at 1:200 dilution induced moderate to strong responses across the four paralogs, reaching 2.9-fold in MgaNR1J1α, 2.3-fold in MgaNR1J1β, 2.1-fold in MgaNR1J1γ, and 3.2-fold in MgaNR1J1δ. At 1:1000 dilution, the activity of *D. labrax* bile decreased markedly and remained below 2-fold in all receptors, with values raging from 1.3-fold to 1.8-fold. Interestingly, *S. aurata* bile showed weaker and more selective activity than *D. labrax* bile. At 1:200 dilution, *S. aurata* bile induced 2.0-fold activation in MgaNR1J1α, 1.6-fold in MgaNR1J1β, 1.9-fold in MgaNR1J1γ, and 2.4-fold in MgaNR1J1δ, whereas at 1:1000 dilution the responses remaining around 1.2-fold and 1.4-fold. Overall, MgaNR1J1δ displayed the strongest bile-induced response in mussel receptors, whereas MgaNR1J1β was the least responsive.

In *R. decussatus* (Figure 5E–H), *D. labrax* bile at 1:200 dilution also displayed the highest responses, reaching 5.2-fold in RdeNR1J1α, 2.5-fold in RdeNR1J1β, 2.4-fold in RdeNR1J1γ, and 3.9-fold in RdeNR1J1δ. At 1:1000 dilution, activity decreased substantially to weak or non-activation (1.9-fold and 1.3-fold). *S. aurata* bile showed a narrower activity profile, inducing 2.9-fold activation in RdeNR1J1α, 1.6-fold in RdeNR1J1β, 2.6-fold in RdeNR1J1γ, and 2.7-fold in RdeNR1J1δ at 1:200 dilution, whereas at 1:1000 dilution, responses returned close to baseline. Overall, these results indicate that clam NR1J1 receptors were more responsive than mussel receptors to fish bile samples, particularly in the case of RdeNR1J1α and RdeNR1J1δ.

## 3. Discussion

NRs are central components of animal chemical-sensing networks because ligand-dependent changes in transcription factor activity can coordinate downstream genes involved in transport, biotransformation, and metabolic homeostasis. In vertebrates, NR1I receptors, particularly PXR and CAR, are major xenobiotic sensors with broad ligand promiscuity and pleiotropic metabolic functions [44,45,46,47]. Comparative studies have further shown that the repertoire of NR1I-related receptors is evolutionary dynamic, being shaped by duplication, loss, and lineage-specific retention across metazoans [48,49,50]. In this context, the protostome NR1J group has emerged as the most plausible evolutionary counterpart of vertebrate NR1I, but functional data remain scarce for this NR group.

In this study, we identified and isolated four *nr1j1* paralogs from the European clam *R. decussatus* and integrated phylogenetic, domain identity, tissue distribution, and transactivation analyses to investigate their relationship with previously characterized mussel NR1J1 receptors. The phylogenetic analysis showed that NR1J1 receptors from the sampled protostome taxa form a well-supported clade that is clearly separated from NR1H, NR1M, and NR1N, while remaining associated with vertebrate NR1I and crustacean NR1L receptors [26,27,40,51]. This pattern is consistent with previous proposals that protostome NR1J1 receptors are part of a broader xenobiotic signaling pathway. This pattern is consistent with previous proposals that NR1J1 receptors are part of a broader xenobiotic signaling pathway [26,27,30,52]. Beyond phylogenetic clustering, the domain-level comparisons further revealed a characteristic NR pattern, namely, higher conservation of the DBD than of the LBD in comparisons between human NR1I and bivalve NR1J1 receptors. Such asymmetry is expected from the modular architecture of NRs, in which the DBD is usually constrained by the need to preserve zinc-finger structure and transcriptional interfaces [53,54], whereas the LBD remains more permissive to diversification in ligand recognition and co-regulator interactions [55,56,57]. The higher similarity observed between orthologous paralogs than between different paralog clades indicates that the α, β, γ, and δ receptors represent evolutionarily stable paralog lineages rather than recent species-specific variants in bivalves [55]. Interestingly, bivalve NR1J1 LBDs remain overall more similar to human VDR (NR1I1) and PXR (NR1I2), suggesting that this domain retains a broadly conserved VDR/PXR-like structural framework [49,57]. In contrast, the DBDs showed paralog-dependent similarity patterns relative to human NR1I receptors, with NR1J1α more similar to CAR (NR1I3), NR1J1β to VDR, and NR1J1γ/δ to both PXR and CAR, indicating that some degree of divergence has also affected the DNA-recognition module. This result is noteworthy because it suggests that, in bivalve NR1J1 receptors, diversification may not be restricted to ligand-related features but may also involve changes in the transcription factor interface that determines response-element recognition and regulatory specificity [54,55]. Accordingly, the matrix is more consistent with a scenario in which *nr1j1* paralogs retained a common ancestral NR1I-like receptor while accumulating clade-specific modifications in both ligand-responsive and DNA-recognition properties, rather than evolving as direct functional equivalents of vertebrate VDR, PXR, or CAR [49,57]. This hypothesis also aligns with the broader view that NR evolution proceeds through stepwise molecular tinkering, in which receptor duplicates preserve the family’s core architecture while exploring new regulatory and functional space through subtle domain-level modifications [55]. Therefore, the domain identity data support the hypothesis that the functional divergence observed in the transactivation assays may reflect not only variation in ligand responsiveness, but also differences in the transcriptional logic of individual NR1J1 paralogs.

The broad tissue distribution of *nr1j1* transcripts in both bivalves indicates that these receptors are not restricted to a specialized detoxification organ. The expression pattern across gill, digestive gland, mantle, adductor muscle, and foot is compatible with functions that extend beyond a narrowly localized xenobiotic-response role [29]. In particular, expression in gill and digestive gland is consistent with participation in environmental sensing and biotransformation-related processes, whereas expression in mantle, foot, and adductor muscle suggests that NR1J1 signaling may also contribute to more widely distributed homeostatic or endogenous regulatory functions [58]. At the same time, the variation in signal intensity among tissues and individuals is consistent with paralog-specific regulation following gene retention and divergence, rather than with complete functional redundancy [59]. Together, these findings support a model in which retained *nr1j1* paralogs preserve related molecular properties while differing in regulatory deployment and physiological context. Nevertheless, the present data support pleiotropic potential, but by themselves do not demonstrate tissue-specific function or downstream target regulation.

The transactivation assays further showed that bivalve NR1J1 receptors are chemically responsive, but not in a functionally uniform manner. Several vertebrate NR1I/NR1H confirmed ligands, particularly GW4064, 24(S),25-epoxycholesterol, allocholic acid, androstenedione and β-estradiol, activated multiple NR1J1 paralogs. These results support the broader concept that evolutionarily related NR1 receptors can exploit overlapping, yet non-equivalent, ligand repertoires through ligand-binding site modifications, as shown for PXR- and liver X receptor (LXR)-related receptors [57,60,61]. Also, the results showed that activation amplitude and receptor sensitivity vary markedly among paralogs and between species. Accordingly, the transactivation profiles are more consistent with a chemically permissive and diversified receptor family than with a single conserved xenobiotic sensor. The marked variability among α, β, γ, and δ paralogs is particularly important from an evolutionary perspective. Such divergence is consistent with evolutionary fates commonly observed after receptor duplication, including retention of overlapping functions together with shifts in tissue distribution, regulatory control, and ligand sensitivity. Comparable scenarios have been discussed for duplicated NRs in vertebrates, including peroxisome proliferator-activated receptor gamma (PPARγ) paralogs retained in some teleost lineages, where different tissue distributions coexist with partially overlapping ligand-response profiles [59], and for LXR- or PXR-related receptors in which lineage-specific retention or loss has been accompanied by functional innovation [49,60]. Moreover, the combined phylogenetic, domain identity, tissue distribution, and transactivation data are consistent with functional divergence after paralog retention. Instead of remaining fully redundant, NR1J1 paralogs may have undergone subfunctionalization or neofunctionalization, leading to differences in regulation and ligand responsiveness. In this context, the broad but variable tissue distribution of *nr1j1* transcripts, together with the paralog-specific sequence similarity patterns and distinct transactivation profiles, supports the hypothesis that these receptors may have diversified their physiological roles. In bivalves, such divergence may have been shaped by adaptation to distinct endogenous and environmental chemical landscapes, including diet-derived compounds and other naturally occurring ligands during evolution [55,57,62,63]. Natural compounds are especially attractive in this context as they may modulate detoxification pathways without the drawbacks associated with prolonged exposure to toxic compounds. In vertebrates, several phytochemicals act on PXR and related xenobiotic-responsive receptors, thereby altering the expression of downstream enzymes and transporters [31,32,33,34,35]. In bivalves, previous studies have mainly focused on downstream detoxification markers (the transcription factor AhR, phase I and phase II detoxification enzymes (CYP and GST genes) and ABC transporters) where compounds such as curcumin, cinnamaldehyde, DL-α-tocopherol, and colchicine were associated with their gene expression and reduced marine toxin accumulation [36,37,64]. The present study provides an upstream receptor-level perspective by directly interrogating NR1J1 transactivation. This is particularly relevant because receptor activation may represent an earlier regulatory step in chemical response pathways. Among the tested natural compounds and extracts, allocholic acid emerged as the most consistent agonist across MgaNR1J1 and RdeNR1J1 paralogs, followed by curcumin, carnosic acid, *P. olacoides* extract, and *D. labrax* bile. Allocholic acid is a naturally occurring bile acid reported in vertebrates [41,42], and bile salts are well-known modulators of vertebrate farnesoid X receptor (FXR), VDR, PXR, and CAR receptors [14,43,57,65,66,67,68]. The strong and sustained activity of allocholic acid, including at low concentrations (25 μM), together with the activity detected in *D. labrax* bile samples, indicates that sterol-derived molecules constitute a productive molecular library for probing bivalve NR1J1 function. A detailed characterization of the fish bile samples would provide further insights into the active principles responsible for NR1J1 activation. Nonetheless, the responses induced by *D. labrax* bile suggest species-specific differences in bile composition that deserve targeted metabolite profiling. Considering that bivalves do not synthesize bile acids and therefore do not require regulation of this metabolic process, the activation of NR1J1 receptors may reflect an evolutionary conserved function retained from the common ancestral receptor. Additionally, receptor activation by bile acids may result from their structural similarity to other environmental relevant compounds, allowing them to interact with the LBDs. Curcumin is a polyphenolic compound that acts as an agonist of the human PXR and has been extensively studied for its therapeutic potential, including antioxidant and anti-inflammatory properties [69,70,71]. The ability of curcumin to modulate MgaNR1J1 and RdeNR1J1 transactivation is consistent with a previous report that showed the modulation of transcriptional factors in mussels by this compound [37]. On the other side, carnosic acid, a phenolic diterpene mainly found in the leaves of Rosmarinus officinalis, has been studied for its anti-inflammatory, antioxidant and neuroprotective properties [72,73]. Seow & Lau (2017) [33] reported a dose-dependent activation of human and mouse PXR by carnosic acid and other chemical constituents of R. officinalis, but not in rat PXR. Interestingly, we identified carnosic acid as a moderate agonist compound of almost all MgaNR1J1 paralogs, again pointing differential receptor sensitivities. Several medicinal plants, such as *P. nigrum*, *Apium graveolens*, and *Hypericum perforatum*, have also been reported as human PXR agonist [35,74]. In this study, the ethanolic extract of *P. olacoides* roots was the only evaluated plant extract that revealed moderate to potent agonist activity on bivalve NR1J1 receptors, MgaNR1J1 being more sensitive than RdeNR1J1 paralogs. These findings suggest that this ethanolic extract may contain one or more bioactive compounds that selectively activate NR1J1 paralogs, although responsible molecules remain unknown. Remarkably, the flavonoid compound chrysin, reported as a human PXR activator [31,75], reduced the activity of all MgaNR1J1 and RdeNR1J1 paralogs, suggesting this compound may act as a potential negative modulator of bivalve NR1J1 receptors. Further research is required to confirm this hypothesis and assign it as inverse agonist of these receptors. Our study defines a first set of chemical probes for dissecting NR1J1 receptor function. These findings highlight the need to explore how natural compounds might regulate marine toxin accumulation or depuration through NR1J1-mediated pathways, enabling the development of innovative strategies to enhance toxic compound metabolism in bivalves. The retention of NR1J1 paralogs with partially overlapping but distinct ligand sensitivities may increase the chemical-response flexibility of bivalves and could facilitate adjustment to species-specific environmental and dietary chemical landscapes. However, this possible adaptive role remains to be tested experimentally.

Taken together, our results suggest that bivalve NR1J1 receptors form a diversified chemical-sensing system with paralog-specific properties, rather than a functionally uniform xenobiotic sensor. The combination of conserved phylogenetic structure, broad tissue distribution, and differentiated transactivation responses indicates that paralog retention in bivalves has been accompanied by functional partitioning at the ligand-response level. From an applied perspective, the identification of non-toxic or less-toxic candidate agonists provides a starting point for testing whether NR1J1 activation is linked to downstream detoxification genes and, ultimately, to toxin-metabolism or depuration performance in vivo. Such validation is now essential, since responses inferred from model vertebrate species cannot be directly extrapolated to predict invertebrate receptor behavior [76].

These findings support future work on the relevance of NR1J1 receptors for comparative toxicology and mechanistic environmental monitoring in bivalves by providing a framework for selecting receptor-specific chemical probes, comparing chemical sensitivity among bivalves, and linking receptor activation to downstream detoxification pathways. Moreover, NR1J1 receptors may become useful mechanistic biomarkers for evaluating chemical exposure and biological response in *M. galloprovincialis*, *R. decussatus*, and other bivalve mollusks if future in vivo studies validate the association between receptor activation, target-gene regulation, and contaminant metabolism.

## 4. Materials and Methods

### 4.1. Sampling, RNA Extraction, and cDNA Synthesis

Five European clams *R. decussatus* and five Mediterranean mussels *M. gallloprovincialis* were purchased at the local market (Docapesca, Matosinhos, Portugal), and no inclusion or exclusion criteria were established. For tissue distribution analyses, gill, digestive gland, mantle, adductor muscle, and foot were dissected from all individuals of each species, preserved in RNAlater, and stored at −80 °C until processing. Total RNA was extracted from all tissues following the protocol described by Casas-Rodríguez et al. (2024) [30]. RNA quantity and purity were assessed using DeNovix DS-11 FX spectrophotometer (DeNovix Inc., Wilmington, DE, USA). RNA extraction from the adductor muscle of *R. decussatus* individual 5 failed, so this sample was not included in the further experiments. First-strand cDNA was synthesized from 250 ng of total RNA using the NZY First-Strand cDNA Synthesis Kit (NZYTech, Lisbon, Portugal), according to the manufacturer’s instructions. For the isolations of *R. decussatus nr1j1* paralogs, 5′- and 3′-RACE cDNA was synthesized from gill RNA using the SMARTer RACE cDNA Amplification kit (Takara Bio, Kusatsu, Japan), following the manufacturer’s guidelines.

### 4.2. Isolation of R. decussatus nr1j1 Paralogs

Partial sequences corresponding to *R. decussatus nr1j1* paralogs were first identified by tBLASTn (v2.12.0) searches performed against the NCBI SRA, using previously characterized *M. galloprovincialis nr1j1* paralogs as query sequences [30]. The transcriptomic datasets SRX171153 to SRX171164 were explored for the identification of the *nr1j1α* and *nr1j1β* paralogs and SRX171153 to SRX171176 for the *nr1j1γ* and *nr1j1δ* paralogs. The retrieved partial sequences were assembled from the SRA data in Geneious^®^ (v7.1.7, Biomatters, Auckland, New Zealand) and used to design gene-specific primers for rapid amplification of cDNA ends (RACE) and subsequent open reading frame (ORF) confirmation. Because the assembled transcript sequences were incomplete, 5′- and 3′-RACE were performed to recover the missing regions. Full ORFs were then amplified by PCR using primers designed on the start codon and stop codon positions to confirm the complete coding sequence. PCR products were cloned into pGEM-T Easy Vector System (Promega, Madison, WI, USA) prior to Sanger sequencing (Eurofins GATC, Constance, Germany). Primer sequences and PCR conditions used with the Phusion Flash High-Fidelity PCR Master Mix (Thermo Fisher Scientific, Waltham, MA, USA) are provided in Supplementary Table S2.

### 4.3. Phylogenetic Analysis

Amino acids sequences of NR1I and NR1H receptors from deuterostomes and NR1J, NR1H, NR1L, NR1M, and NR1N receptors from protostomes were retrieved from public databases, including NCBI GenBank and the Joint Genome Institute (JGI) database for *Lottia gigantea* (G. B. Sowerby I, 1834). The complete list of sequences used is provided in Supplementary Table S3. Multiple sequence alignment was performed with MAFFT v7 using the L-INS-i method. The final alignment comprised 73 taxa and 1705 amino acid positions. Maximum-likelihood tree inference was performed with PhyML 3.0 (LIRMM, Montpellier, France), using SMS to automatically select the best-fitting substitution model, which in this case was Q.insect +R+F. Branch support was assessed using the approximate Bayes, aBayes, procedure implemented in PhyML 3.0 (LIRMM, Montpellier, France) (Figure 1A). NR1M and NR1N sequences were used as outgroups to root the tree.

### 4.4. Tissue Distribution Analysis

The tissue distribution of *nr1j1* paralogs was assessed by endpoint PCR using cDNA synthesized from gill, digestive gland, mantle, adductor muscle, and foot of five individuals of *M. galloprovincialis* and *R. decussatus*. PCR reactions were performed in 10 μL final volume using NZYTaq II 2× Green Master Mix (NZYTech, Lisbon, Portugal) and gene-specific primers listed in Supplementary Table S2. Amplification products were separated in 2% agarose gels. The ribosomal genes 18S and 28S were used as reference markers to verify cDNA quality and amplification consistency across tissues.

### 4.5. Chemical and Reagents

Rifampicin (CAS: 13292-46-1) were bought from Fisher BioReagents, Pittsburgh, PA, USA. Dexamethasone (CAS: 50-02-2), GW4064 (CAS: 278779-30-9), lanosterol (CAS: 79-63-0), cholesterol (CAS: 57-88-5), chenodeoxycholic acid (CAS: 474-25-9), testosterone (CAS: 58-22-0), and androstenedione (CAS: 63-05-8) were acquired from Sigma-Aldrich, Saint Louis, MO, USA. Allocholic acid (CAS: 2464-18-8), glabridin (CAS: 59870-68-7), alisol B 23-acetate (CAS: 26575-95-1), cafestol (CAS: 469-83-0), and nomilin (CAS: 1063-77-0) were purchased from MedChemExpress, Monmouth Junction, NJ, USA. Isorhamnetin (CAS: 480-19-3) was obtained from TCI EUROPE, Zwijndrecht, Belgium. 8-methoxypsoralen (CAS: 298-81-7), carnosic acid (CAS: 3650-09-7), and DMSO (67-68-5) were bought from Merck, Darmstadt, Germany. SR12813 (CAS: 126411-39-0), cholic acid (CAS: 81-25-4), lithocholic acid (CAS: 434-13-9), 25-hydroxycholesterol (CAS: 2140-46-7), 24(S)-Hydroxycholesterol (CAS: 474-73-7) were acquired from Tebu-Bio, Le Perray-en-Yvelines, France. Chrysin (CAS: 480-40-0), 3-hydroxyflavone (CAS: 577-85-5), and curcumin (CAS: 458-37-7) were purchased from Thermo Fisher Scientific, Waltham, MA, USA. 17β-estradiol (CAS: 50-28-2) were obtained from Santa Cruz Biotechnology, Dallas, TX, USA. 24(S),25-Epoxycholesterol (CAS: 77058-74-3) were bought from Cayman Chemical, Ann Arbor, MI, USA. Taxol (CAS: 33069-62-4) was acquired from Focus Biomolecules, Plymouth Meeting, PA, USA. Okadaic acid (CAS: 78111-17-8) was obtained from Enzo Life Sciences, Farmingdale, NY, USA. Confirmed ligands of the vertebrate NR1I and NR1H receptors were selected to test possible functional conservation or overlap with bivalve NR1J1 receptors. Natural compounds were selected as potentially less toxic modulators of NR1J1 receptors based on a literature review of studies assessing potential agonist compounds of human PXR.

### 4.6. Plant Extracts

The dried plant materials of *Pueraria lobata* (Root), *P. olacoides* (Root), *P. nigrum* (Fruit), *Angelica archangelica* (Root), and *A. graveolens* (Seed) were purchased from a local Portuguese supplier (Ervanário Portuense, Porto, Portugal). The fresh material of *C. esculentus* (Root) was obtained from the Botanical Garden of the University of Porto and freeze-dried using LyoQuest (Telstar, Terrassa, Spain). To obtain the plant extract, 5 g of each dried plant material was macerated and subjected to extraction using 95% ethanol in an automated Accelerated Solvent Extractor (Dionex™ ASE™ 350, Thermo Fisher Scientific, Waltham, MA, USA) at 80 °C, with three extraction cycles of 10 min each and a rinse volume of 100%. The extracts were completely dried by evaporation under reduced pressure and stored at −20 °C until further use. Plant extracts were selected as less-toxic candidate modulators of NR1J1 receptors with possible applied relevance. Particularly, these plant extracts were selected based on a bibliographic review of studies evaluating potential human PXR agonist compounds.

### 4.7. Fish Bile Collection and Preparation

Bile acids were obtained from the fish species European seabass *D. labrax* (Linnaeus, 1758) and gilthead seabream *S. aurata* (Linnaeus, 1758). Bile was collected by piercing the gall bladder with a needle and gently extracting all the content [77]. The extraction was performed in a clean and disinfected area to minimize the risk of contamination. The collected bile was stored at −20 °C until further use. Protein content in the bile acids was relatively quantified using absorbance at 280 nm on DeNovix DS-11 FX spectrophotometer (DeNovix Inc., Wilmington, DE, USA). No extinction coefficient was used. Absorbance values from a curve of Bovine Serum Albumin (BSA) at concentrations of 0.125, 0.25, 0.50, 0.75, and 1 µg/mL were measured as relative indicators of protein content. Fish bile was tested as allocholic acid emerged as an active compound, and bile is a biologically relevant sterol-rich mixture.

### 4.8. Compound Preparation and Testing Concentrations

Stock solutions of the confirmed ligands of NR1I and NR1H receptors were prepared in DMSO at concentrations corresponding to 1000× the tested concentration in the assay. Similarly, the remaining evaluated compounds and plant extracts were dissolved in DMSO at 750× the tested concentration. Work solutions were prepared by diluting the stock solution in DMEM without phenol red (PAN-Biotech, Aidenbach, Bayern, Germany), supplemented with 10% of charcoal-treated fetal bovine serum (PAN-Biotech, Aidenbach, Bayern, Germany) and 1% of penicillin/streptomycin. Final concentrations ranged from 100 to 10 μM for the commercial compounds and from 100 to 50 μg/mL for the plant extracts. Bile acids were directly diluted 200- and 1000-fold in the previously described DMEM medium.

### 4.9. pBIND Vectors Construction

The hinge region and LBD of the four *R. decussatus nr1j1* paralogs (Rde*nr1j1α*, Rde*nr1j1β*, Rde*nr1j1γ*, Rden*r1j1δ*) were amplified by PCR using Phusion Flash High-Fidelity PCR Master Mix (Thermo Fisher Scientific, Waltham, MA, USA) and the primers listed in Supplementary Table S2. After digestion, the amplified fragments were cloned into the pBIND plasmid (Accession number: AF264722; Promega, Madison, WI, USA) to generate GAL4 DNA-binding domain fusion constructs containing the hinge and the LBD of each receptor [78]. The Rde*nr1j1α* fragment was cloned using XbaI/KpnI, the Rde*nr1j1β* and Rde*nr1j1γ* fragments using BamHI/KpnI, and the Rde*nr1j1δ* fragment using NotI/KpnI. The cloned inserts were confirmed by automated Sanger sequencing (Eurofins GATC, Constance, Germany) and the sequences deposited in GenBank under the accession numbers OR892309.1, OR892310.1, OR892311.1, and OR892312.1. The pBIND plasmids containing the hinge region and LBD of the four *M. galloprovincialis nr1j1* paralogs, as well as human PXR, were kindly provided by Elza Fonseca [30,49].

### 4.10. Cell Culture and Transactivation Assays

COS-1 cells (ATCC^®^ CRL-1650, Manassas, VA, USA) were maintained in DMEM with phenol red (PAN-Biotech, Aidenbach, Bayern, Germany), supplemented with 10% fetal bovine serum (PAN-Biotech, Aidenbach, Bayern, Germany) and 1% penicillin/streptomycin (PAN-Biotech, Aidenbach, Bayern, Germany), at 37 °C with 5% CO_2_ and 95% relative humidity. Cells were cultured following ATCC recommendations. For luciferase-based reporter transactivation assays, COS-1 cells were seeded in 96-well culture plates at a density of 4 × 10^4^ live cells/well. After 24 h, cells were transfected with 25 ng/well of the evaluated pBIND construct and 50 ng/well of pGL4.35 [luc2P/GAL4UAS/Hygro] luciferase reporter vector (Promega, Madison, WI, USA), with lipofectamine 2000 reagent (Invitrogen, Carlsbad, CA, USA) in Opti-MEM reduced-serum medium (Gibco, Carlsbad, CA, USA), following the manufacturer’s indications.

After 5 h of incubation, the transfection medium was removed, and cells were exposed to the tested compounds. Each assay included okadaic acid (50 nM) and DMSO (<0.15%) as positive and solvent control, respectively. After 24 h of exposure, cells were lysed, and Firefly and Renilla luciferase activities were determined using the Dual-Luciferase^®^ Reporter Assay System (Promega, Madison, WI, USA), according to the manufacturer’s instructions. Luminescence was measured using a Synergy HT Multi-Mode Microplate Reader (Biotek, Agilent, Santa Clara, CA, USA). Each condition was tested with two technical replicates in three independent assays (n = 3). Transactivation results are expressed as fold induction, calculated as the ratio of firefly and Renilla luciferase activities in treated cells and normalized to the corresponding solvent control.

### 4.11. Statistical Analysis

Statistical analyses were applied for the functional screening of confirmed ligands of vertebrate NR1I and NR1H receptors, dose–response assays and bile acid evaluation. The normality of data distribution and homogeneity of variances were assessed using Shapiro–Wilk test and Levene’s test, respectively. Non-parametric analyses were performed using the Kruskal–Wallis test, followed by multiple comparisons corrected for false discovery rate (FDR) using the Benjamini–Hochberg method. All analyses were conducted using GraphPad Prism (v11.0.0, GraphPad Software, Boston, MA, USA, www.graphpad.com). A q-value < 0.05 was considered statistically significant. Data are presented as mean ± standard error of the mean (n = 3). Detailed statistical analysis, including raw data, Kruskal–Wallis test outputs, and pos-hoc multiple comparison results are provided in Supplementary Table S4.

## 5. Conclusions

This study shows that NR1J1 receptors from *M. galloprovincialis* and *R. decussatus* display distinct but partially overlapping transactivation profiles in response to confirmed ligands of vertebrate NR1I and NR1H receptors, sterol-derivates, natural compounds, plant extracts, and fish bile samples. Together with the phylogenetic, domain identity, and tissue distribution analyses, these results reinforce the idea that bivalve NR1J1 receptors constitute a diversified chemical-sensing system instead of a single functionally uniform xenobiotic sensor. These findings further suggest that bivalve sensing mechanisms may have evolved under different environmental and metabolic pressures, contributing to functional diversification. The identification of active natural compounds and sterol-rich bile samples further highlights NR1J1 receptors as promising candidates for future studies on ligand-dependent regulation and detoxification-related pathways in bivalves. Further research is needed to determine whether NR1J1 activation is linked to downstream gene regulation and to measurable effects on toxin handling or depuration in vivo.

## Data Availability

The original contributions presented in this study are included in the article/Supplementary Materials. Further inquiries can be directed to the corresponding author.

## Supplementary Materials

The following supporting information can be downloaded at: https://www.mdpi.com/article/10.3390/ijms27156810/s1.

## Abbreviations

The following abbreviations are used in this manuscript:

NR1J1	Nuclear receptor 1 subfamily J1
NR	Nuclear receptor
PXR	Pregnane X receptor
CAR	Constitutive androstane receptor
SRA	Sequence Read Archive
RACE	Rapid amplification of cDNA ends
JGI	Joint Genome Institute
LBD	Ligand-binding domain
DBD	DNA-binding domain
FDR	False discovery rate
LXR	Liver X receptor
VDR	Vitamin D receptor
PPARγ	Peroxisome proliferator-activated receptor gamma
ORF	Open reading frame
