# Supplementary material for: Functional and Evolutionary Insights of NR1J1 Nuclear Receptors: A Diversified Sensing Weapon in Bivalves
Source: Int J Mol Sci. 2026 Jul 29;27(15):6810. doi: 10.3390/ijms27156810 (PMC13467592; doi:10.3390/ijms27156810)

Supplementary Figure S1. Transactivation responses of human PXR following exposure to (**A**) confirmed ligands of vertebrate NR1I and NR1H receptors, (**B**) selected natural compounds and plant extracts at the highest tested concentration, (**C**) selected natural compounds and plant extracts at the indicated concentrations, and (**D**) fish bile samples from at two dilutions. Significant differences relative to the DMSO control are represented by * q-value < 0.05, ** q-value < 0.01 (n = 3).

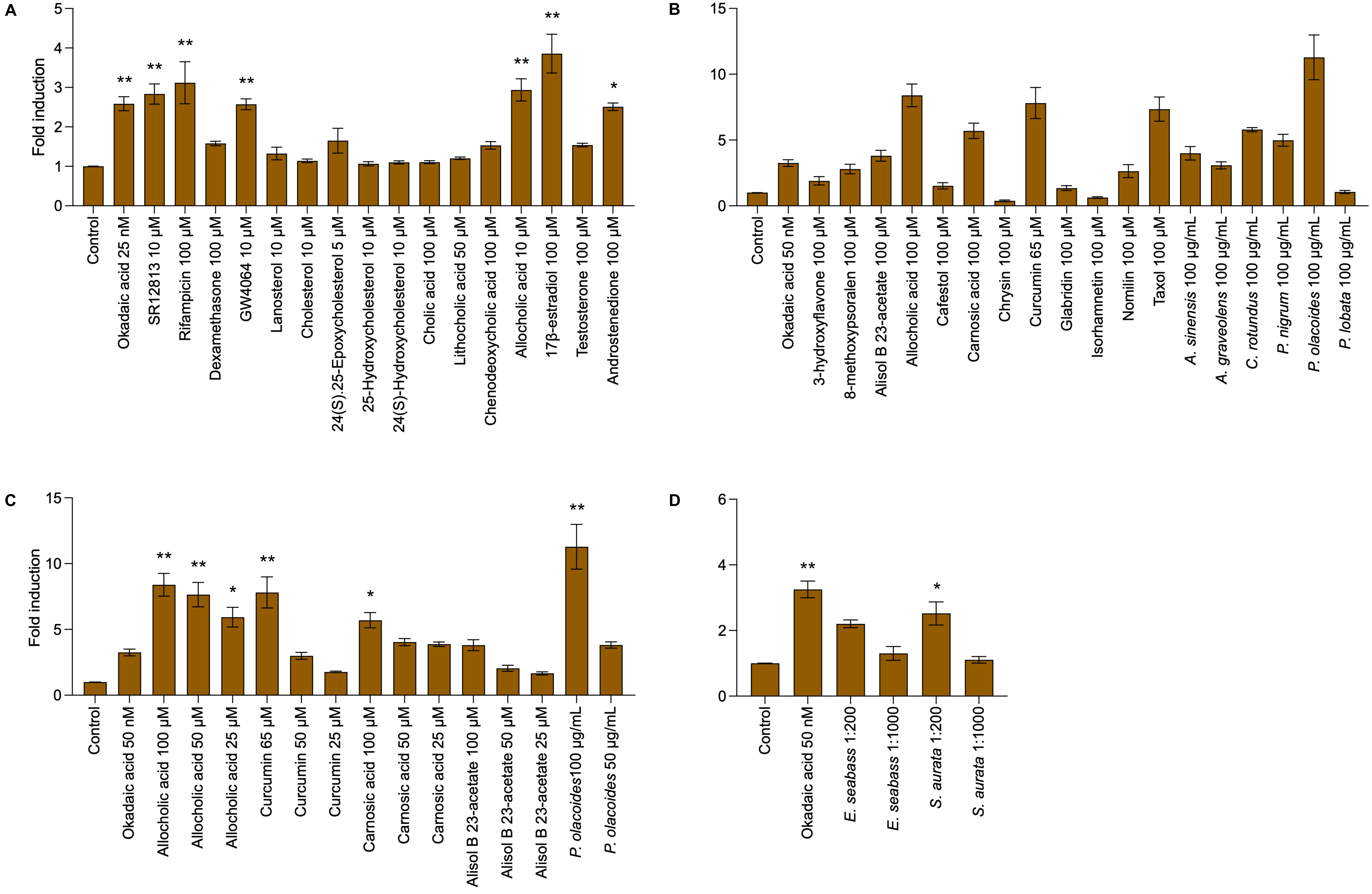

Supplement: Supplementary file 1 [file ijms-27-06810-s001.zip › SupplemendaryFigure_S1.pdf]
